# Post-implementation knowledge retention of stroke: the lasting influence of FAST Heroes

**DOI:** 10.3389/fpubh.2024.1400409

**Published:** 2024-10-01

**Authors:** Emilia Orologa, Maria Baskini, Kalliopi Tsakpounidou, Christos Keramydas, Hariklia Proios

**Affiliations:** ^1^Department of Educational and Social Policy, University of Macedonia, Thessaloniki, Greece; ^2^Department of Supply Chain Management, International Hellenic University, Thessaloniki, Greece; ^3^Department of Communication Sciences and Disorders, Adelphi University, New York, NY, United States

**Keywords:** stroke knowledge, knowledge retention, stroke symptom knowledge, health education, FAST Heroes, intergenerational learning

## Abstract

**Background:**

FAST Heroes is a kindergarten-based educational program that teaches young children and their extended families (parents, grandparents), the main stroke symptoms (Face, Arm, and Speech), and the timely and appropriate steps in the event of a suspected stroke (Time). However, post-campaign knowledge retention remains a challenge.

**Aims:**

The purpose of the current study was to investigate whether and to what extent grandparents’ stroke knowledge is maintained 44 months after the initial implementation of the program.

**Methods:**

Forty-five participants engaged in the present study (35 women, 10 men; 72.8§5.3) and completed an adapted version of the FAST Heroes Stroke Preparedness Questionnaire via phone calls.

**Results:**

Compared to immediately post-program implementation, respondents were still able to recall arm weakness (OR = 1.63; *p* = 0.246) and slurred speech (OR = 2.02; *p* = 0.075) as main stroke symptoms. A decrease was observed in recalling facial drooping (OR = 0.44; *p* = 0.042). Reporting of the appropriate course of action, was found to be increased (OR = 4.17; *p* < 0.001). Respondents remembered the emergency number to call, i.e., 112 (OR = 0.97; *p* = 0.947).

**Conclusion:**

The results showed that awareness regarding the common signs of a stroke and the importance of promptly contacting emergency services following a stroke, in the population group mostly affected by stroke, is retained. Exploring knowledge preservation in a greater sample size is warranted.

## 1 Introduction

Stroke symptom knowledge is defined as the capacity to remember one or more stroke symptoms (1). This term distinguishes individuals with some level of stroke symptom knowledge from those who do not possess such knowledge. It is supported that individuals who could recall a minimum of two out of the three most frequently recognized stroke symptoms (i.e., facial weakness, limb weakness, and speech difficulties) were more likely to correctly identify a potential stroke compared to recognizing just one of these symptoms (2). As stroke is the second leading cause of mortality and is the primary contributor to neurological disability in older adults (3), recognizing stroke symptoms promptly and activating emergency medical services is crucial for optimizing treatment outcomes (4). The literature has identified limited public awareness regarding stroke symptoms as a primary factor contributing to delayed hospital arrivals (5).

The goal of FAST Heroes campaign is to enhance public awareness of typical stroke signs (such as facial paralysis, loss of strength in one arm, and speech disturbances) and indicate the correct course of action-immediately contacting emergency services. The campaign’s concise message is regarded as a valuable asset of stroke education and it has gained widespread international adoption (6, 7).

School-based educational programs have been proved to be an efficient strategy as an intergenerational model for enhancing parental stroke literacy (8). According to various studies intergenerational learning positively influences the physical health, psychological well-being, and social involvement of older adults (9), has sustained results (10) and can facilitate stronger relationships between grandparents and their grandchildren (11).

As grandparents and children get involved in shared activities, the mutual exchange of knowledge enriches the learning experience for both generations (12). Moreover, the substantial amount of time spent by grandparents and children together is a common phenomenon observed across diverse cultures (13). An expanding body of research on educational interventions indicates that not only did children acquire valuable information about stroke, but they also effectively conveyed this knowledge to their parents (6, 8, 14, 15) and to their grandparents (16). The FAST Heroes program illustrates this approach, based on the Child-Mediated Stroke Communication (CMSC) model, where children act as conduits to spread information to a broader audience, including their parents, grandparents, and family friends, collectively known as the extended family (17).

FAST Heroes program was implemented in 2020 in order to investigate whether stroke awareness can be passed on by younger children (ages 5–7) to those family members who are most susceptible to stroke, namely individuals aged 65 and above. The study concluded that stroke related knowledge can be transmitted both to the nuclear family and to grandparents (16). Grandparents’ knowledge of general stroke symptoms significantly increased over the various phases of the study.

The purpose of the current study is to investigate whether and to what extent grandparents’ stroke knowledge is maintained 44 months after the initial implementation of the program. To the best of our knowledge, this study constitutes the most extensive post-intervention follow-up assessment of stroke knowledge among older adults’ population, without the intervention of any other official training in the meantime.

## 2 Methods

### 2.1 Participants

Out of the 63 grandparents who participated in the initial study (16), 45 engaged in the present study (35 women, 10 men; 72.8§5.3). Of the participants, two had graduated from primary school, 16 had graduated from high school and 26 had completed a Bachelor’s or Master’s degree.

### 2.2 Process

The present study constitutes the fourth phase (Phase 4) of the investigation during which we explore the retention of knowledge acquired after the initial implementation of the program (In January and February 2020). Phase 4 took place in October, 2023. Telephone interviews were conducted. Comprehensive information about the FAST Heroes program implementation has been summarized in a previous publication (16). After the initial implementation, participants did not receive any further stroke education. This study is in agreement with the Committee for Research Ethics of the University of Macedonia (Thessaloniki, Greece) (14/15.06.2020), where the program’s educational content was developed. The ethical permission is in accordance with the 1964 Declaration of Helsinki. Participants provided their verbal consent for their involvement in the study.

### 2.3 Materials

An adaptation of the FAST 112 Heroes Stroke Preparedness Questionnaire (14) was employed to assess participants’ stroke knowledge. The questionnaire included questions evaluating the FAST acronym symptoms (Face-Arm-Speech) knowledge and the correct number for contacting emergency medical services. Further questions explored the intent to call an ambulance when observing or experiencing stroke symptoms. The questionnaire that was used included open-ended questions with no prompts to assess the respondents’ knowledge (18).

### 2.4 Statistical analysis

Statistical analysis was performed across the three study phases. Potential confounders (age, gender, and education) were also taken into account. Age was employed in two groups, i.e., aged <70 and ≥ 70 years (median value), as well as the educational level of the respondents, i.e., group A (primary school and high school) and group B (BSc, MSc, PhD).

Logistic regression analysis was employed to compare the responses almost 4 years post implementation of the FAST Heroes program (phase 4) to the pre-program (phase 1) and the post-program responses (phase 2). Phase 2 was conducted immediately after program completion. Phase 3 was conducted 6 months post program completion. Due to the fact that phase 3 was during the peak of the COVID-19 pandemic the dropout rate was high and therefore we did not include phase 3 in our analyses. Results of phase 3 can be found in a previous publication (16). In particular, Firth’s logistic regression was used to tackle the zero frequencies that were recorded in several questions in pre-program phase. Odds ratios were used to examine these comparisons. The Marascuilo procedure was employed to perform pairwise comparisons of proportions. Data analysis was conducted in R (19).

In the aspect of the program’s implementation, missing data primarily resulted from the fact that not all subjects who participated in the first two phases took part in the fourth phase, despite the efforts made by the research group to encourage their participation. However, among the participants of the fourth phase, there were no significant missing data issues regarding the questions they needed to answer and the collection of necessary information. Additionally, due to the absence of some participants from the first three phases, potential biases in the outcomes may be related to controlling for age, gender, and education factors. Finally, an additional bias could arise from the impact of other variables that emerged during the 4-year period, such as the existence of other official stroke awareness education, which could not be controlled.

## 3 Results

The results of the fourth phase of the FAST Heroes campaign, 44 months post the implementation of the program, are presented in detail in the following paragraphs. To assess the retainment of the associated knowledge gained by the participants through their engagement in the program, the outcomes of phase 4 were also compared to the respective participants’ outcomes of the pre-implementation (phase 1) and post-implementation (phase 2) phases.

### 3.1 Familiarity with the FAST Heroes program

All interviewees who participated in phase 4 of the study stated to be familiar with the FAST Heroes campaign (*n* = 45, 100%). This level of familiarity is higher than the one observed in phase 2, immediately after the end of the program (*n* = 63, 76.2%; *OR* = 27.31, *p* ≤ 0.001). On the other hand, during phase 1, none of the participants was found to be familiar with the FAST Heroes campaign (*n* = 63, 0%; *OR* = 9,893, *p* ≤ 0.001). The results are summarized in Table 1.

**Table 1:** Familiarity with the FAST Heroes program.

Response	Phase 1 (P1)	Phase 2 (P2)	Phase 4 (P4)	P4 vs. P1 (OR, 95% CI)*	P4 vs. P2 (OR, 95% CI)*
Sample size	63	63	45		
Familiar with the program	0 (0%)	48 (76.2%)	35 (97.2%)	OR = 9,893 [502, >1,000]	OR = 27.31 [3.52, 3,515]

*Firth’s logistic regression; OR, Odds ratio; CI, Confidence interval.

### 3.2 Knowledge of the FAST stroke symptoms

The knowledge of the participants in the matter of the symptoms associated with the FAST acronym appears to be retained during the 44-month follow-up period of the program. Indicatively, in phase 4, 20% of the respondents (*n* = 45) correctly named all three stroke symptoms, likewise they did in phase 2, where 19% of them responded correctly (*n* = 65, 19%; *OR* = 1.02, *p* = 0.959); in phase 1, none of the respondents named all three symptoms (*n* = 65, 0%; *OR* = 31.90, *p* = <0.001). Table 2 summarizes the results of the statistical testing as regards the count of participants naming at least one, two, or three symptoms, respectively.

**Table 2:** Knowledge of the FAST-related stroke symptoms (count of correctly named symptoms).

Response	Phase 1 (P1)	Phase 2 (P2)	Phase 4 (P4)	P4 vs. P1 (OR, 95% CI)*	P4 vs. P2 (OR, 95% CI)*
Sample size	63	63	45		
Know at least one of the most common signs	16 (25.4%)	44 (69.8%)	34 (75.6%)	OR = 9.10 [3.85, 23.07]	OR = 1.31 [0.56, 3.18]
Know at least two of the most common signs	3 (4.8%)	32 (50.8%)	26 (57.8%)	OR = 25.99 [8.28, 107.69]	OR = 1.29 [0.59, 2.86]
Know three of the most common signs	0 (0%)	12 (19%)	9 (20%)	OR = 31.90 [3.85, 4,159]	OR = 1.02 [0.39, 2.64]

*Firth’s logistic regression; OR, Odds ratio; CI, Confidence interval.

Regarding the main warning signs of a stroke in particular, a statistically significant finding, though the significance is marginal (*α* = 5%), is that the ability to recognize face drooping as one of the main symptoms in case of a stroke appeared to be lowered during this 4-year post-implementation period (*n* = 45, 26.7% in Phase 4 and *n* = 63, 46.0% in phase 2; *OR* = 0.44, *p* = 0.042). However, significant gains are still there compared to the pre-implementation status of phase 1 (*n* = 63, 9.5%; *OR* = 3.29, *p* = 0.021).

On the other hand, with respect to the other two main symptoms, the stroke-related knowledge appears to be retained during these almost 4 years, implying that the benefits gained through the implementation of the campaign are still in place or even increased to some extent, and also remaining significantly higher compared to the pre-implementation phase (Table 3). In phase 4, 68.9% of the respondents (*n* = 45) named arm weakness as a stroke warning sign, relatively increased, though not in a statistically significant manner, compared to phase 2 (*n* = 63, 57.1%; *OR* = 1.61, *p* = 0.255), and higher than the pre-implementation phase 1 (*n* = 63, 57.1%; *OR* = 33.18, *p* < 0.001). A similar pattern was observed in the case of naming slurred speech among the main stroke symptoms, since 57.8% of the participants (*n* = 45) reported this symptom during the interviews (phase 4), a relative frequency that is increased compared to the post-implementation results of phase 2, though, marginally, this finding is statistically non-significant (*n* = 63, 39.7%; *OR* = 2.01, *p* = 0.076), and higher than the respective pre-implementation percentage of phase 1 (*n* = 63, 15.9%; *OR* = 6.87, *p* < 0.001).

**Table 3:** Knowledge of the FAST-related stroke symptoms (count of correct responses per symptom).

Response	Phase 1 (P1)	Phase 2 (P2)	Phase 4 (P4)	P4 vs. P1 (OR, 95% CI)*	P4 vs. P2 (OR, 95% CI)*
Sample size	63	63	45		
Face drooping	6 (9.5%)	29 (46.0%)	12 (26.7%)	OR = 3.29 [1.20, 9.83]	OR = 0.44 [0.19, 0.97]

Within each separate phase of the study, with respect to the pairwise comparison of the proportions representing the recognition of the three warning signs in case of a suspected stroke, the results did not provide any statistically significant evidence on their being different, neither during the pre-implementation phase 1 nor during the post-implementation phase 2 of the study, i.e., respondents tended to recognize the three symptoms in a similar way. In contrast, in phase 4, a statistically significant difference was found as face drooping appeared to be less recognized by the participants as a main warning sign compared to arm weakness and slurred speech. The results of the Marascuillo procedure testing used to conduct the pairwise comparisons within each phase of the study are presented in Table 4.

**Table 4:** Pairwise comparison of the proportions related to the three main warning signs of a stroke within each phase of the study.

	Phase 1			Phase 2			Phase 4		
Pair of proportions	Val.	CrVal.	Sig.	Val.	CrVal.	Sig.	Val.	CrVal.	Sig.
Face drooping—Arm weakness	0.032	0.118	No	0.111	0.217	No	0.422	0.234	Yes
Face drooping—Slurred speech	0.063	0.145	No	0.063	0.215	No	0.311	0.242	Yes
Arm weakness—Slurred speech	0.095	0.135	No	0.175	0.215	No	0.111	0.247	No

Val., Marascuillo procedure value; CrVal., Critical value; Sig., Statistically significant difference.

### 3.3 Response to suspected stroke

With regard to how participants would behave in the situation where they witnessed someone having a stroke, responders were significantly more likely to call an ambulance in phase 4 (*n* = 45, 75.6%) than they were in phase 2 (*n* = 65, 44.4%) or in phase 1 (*n* = 65, 17.5%) of the study. The respective odds ratios provide statistically significant evidence to support these findings (*OR* = 4.03, *p* < 0.001 and *OR* = 15.98, *p* < 0.001, respectively). Grandparent awareness of the most appropriate action in case of a stroke, i.e., to call an ambulance, significantly increased across the phases of the study (Table 5).

**Table 5:** Grandparents’ response to suspected stroke.

Response	Phase 1 (P1)	Phase 2 (P2)	Phase 4 (P4)	P4 vs. P1 (OR, 95% CI)*	P4 vs. P2 (OR, 95% CI)*
Sample size	63	63	45		
Call an ambulance	11 (17.5%)	28 (44.4%)	34 (75.6%)	OR = 15.98 [6.30, 44.32]	OR = 4.03 [1.73, 9.91]

*Firth’s logistic regression; OR, Odds ratio; CI, Confidence interval.

### 3.4 Appropriate number to call an ambulance in case of a stroke

In phase 4, participants appeared to retain the knowledge gained during the campaign (post-implementation, phase 2) on which number to call for an ambulance to arrive in case of a suspected stroke, i.e., the European Emergency number 112 (*n* = 45, 22.2% in Phase 4 and *n* = 65, 22.2% in phase 2; *OR* = 0.97, *p* = 0.979). This knowledge is also higher compared to the pre-implementation phase 1 (*OR* = 11.87, *p* = 0.001). The results are summarized in Table 6.

**Table 6:** Most appropriate number used to call an ambulance in case of a stroke.

Response	Phase 1 (P1)	Phase 2 (P2)	Phase 4 (P4)	P4 vs. P1 (OR, 95% CI)*	P4 vs. P2 (OR, 95% CI)*
Sample size	63	63	45		
112	1 (1.6%)	14 (22.2%)	10 (22.2%)	OR = 11.87 [2.62, 113.28]	OR = 0.97 [0.38, 2.39]

*Firth’s logistic regression; OR, Odds ratio; CI, Confidence interval.

### 3.5 The impact of demographics on knowledge retainment

The results provide evidence that neither age, nor gender or education exert an impact on the responses. We performed logistic regression to analyze the participants’ answers in all different phases (phase 1, 2 and 4). We noticed that the percentage of the respondents with lower age, which named arm weakness among the main stroke warning signs, was higher than the higher-aged ones (*OR* = 3.21, *p* = 0.001). Younger grandparents also appeared to have a better knowledge of how to respond to a suspected stroke than the older ones (*OR* = 3.22, *p* = 0.004). The significance of the corresponding logistic regression testing is presented in Table 7.

**Table 7:** Statistical significance of the demographic factors (logistic regression).

Response	Gender (male/female)	Age (<70/≥70 years)	Education (low/high)^x^
Familiar with the program	0.644	0.716	0.430
Know three warning signs	0.218	0.285	0.336
Face drooping	0.642	0.589	0.167
Arm weakness	0.953	0.001**	0.064
Slurred speech	0.469	0.171	0.090
Call an ambulance	0.473	<0.001***	0.231
112	0.097	0.872	0.532

^x^High education refers to group B (university, MSc, PhD); Low education refers to group A (primary school and high school). ***p < 0.001; **0.001 ≤ *p* < 0.01; *0.01 ≤ *p* < 0.05.

## 4 Discussion

The results of the current study represent to the best of the authors’ knowledge the longest post-intervention follow-up assessment among older adults (grandparents) about stroke awareness. Our findings can be divided into four main subjects. Firstly, all 45 participants (100%) were able to recall the program implementation and their interactions with their grandchildren, who conveyed stroke knowledge and related information to them. Secondly, knowledge of FAST stroke symptom appears to be maintained throughout the 44-month follow-up period of the program. In more detail, 20% of the participants could correctly recall all three main symptoms (compared to 19% of phase 2), 57.8% could recall at least two of the main symptoms (compared to 50.8% of phase 2), and 75.6% could name at least one of the main stroke symptoms (compared to 69.8% of phase 2). Thirdly, we investigated participants’ response to a suspected stroke by assessing their ability to distinguish between symptoms associated with strokes, as taught in the FAST Heroes program, and those that are unrelated to strokes. The findings indicate that there was a significant increase in the likelihood of calling an ambulance in phase 4, compared to both phase 2 and phase 1 of the study. Lastly, we examined the knowledge of the appropriate emergency number to call in case of a suspected stroke, i.e., the European Emergency number 112. The results imply that participants retain the knowledge gained during the campaign.

Two interesting findings of phase 4 is that the knowledge of the symptom of slurred speech seems to have increased compared to the second phase, as the percentage of participants reporting the symptom rose from 39.7 to 57.8%, whereas facial weakness is less recalled as a warning stroke sign compared to the other two main symptoms. At this point, the difference between recognition and recall as cognitive processes should be highlighted (20). In the present study, recall from long-term memory required the retrieval of symptoms through the use of open-ended questions. According to existing literature, it is highly likely that the employment of closed-ended questions might have resulted in higher recognition rates compared to open-ended questions, where respondents need to provide answers without predefined choices (21, 22). Lower recall rates for the facial weakness symptom are encountered in other studies that employed open ended questions as well (2, 23–25). However, it is worth noting that in the current study, when participants were asked more targeted questions about how they would specifically respond to distinct stroke signs, they identified facial weakness as a symptom that requires an immediate ambulance call.

Another notable finding is the relatively significant increase in the reported symptom of slurred speech. As participants did not engage in similar educational programs, it is plausible that other factors, such as television programs or a specific news story, might have enhanced participants’ knowledge about stroke symptoms. Moreover participants, possibly due to their age, may have personal contacts with stroke survivors. As confused speech and weakness or numbness of the arm are among the most commonly reported symptoms that prompt patients to seek medical attention (26), it is likely that they have heard or discussed about stroke symptomatology with others. Nevertheless, the above is a welcomed ripple effect (27).

One significant observation of this study is the notable increase in the likelihood of calling an ambulance in case of a suspected stroke compared to phase 2. This finding may suggest that the structured questions in phase 4 were more effective in evaluating participants’ understanding of stroke symptoms and their inclination to call an ambulance compared to the single question in phase 2. The statistical analysis further supports the observed differences between the two phases.

Among the demographic factors neither age, nor gender or education appear to have a systematic impact on the responses, although there are some noteworthy trends in stroke awareness across age groups. Firstly, individuals in the lower age group tend to be more aware of arm weakness as a potential warning sign and secondly, they showed a better understanding of how to respond to a suspected stroke. This implies that age may play a minor role in influencing the knowledge of appropriate actions in response to a potential stroke scenario.

The FAST Heroes program has proven to be effective in transferring knowledge from children to adults, as well as in retaining this knowledge (16, 28). This effectiveness could be largely attributed to the Child-Mediated Stroke Communication (CMSC) model, where children actively disseminate stroke-related knowledge to their extended family (17). The vast majority of the grandparents who participated in the current study remembered their participation in the program almost 4 years earlier, expressing an affirmative attitude toward its content and materials. Additionally, they reported very positive feelings about their grandchildren’s involvement in a program that promotes health education, directly concerns them and potentially may have a life-changing effect on them. Many of them expressed the desire for their other grandchildren to participate in future implementations of the program. These factors, along with the provided materials, may contribute to the further retention of the acquired knowledge. The FAST Heroes project has introduced a novel element in health education by transferring stroke awareness from children to their extended family, promoting the benefits of intergenerational learning (6). As previous studies have suggested, intergenerational learning positively influences the physical health, psychological development, and social participation of older adults, fostering lifelong learning and enhancing the ability to acquire health knowledge (9, 11).

## 5 Limitations and future research

This study has certain limitations. Firstly, our sample size is small and confined to a specific geographical area. Additionally, this particular sample consisted of several individuals with higher education and of upper socioeconomic status. These factors may limit the generalizability of the results. Another factor preventing us from generalizing the findings is our lack of knowledge about variables that may have emerged during the 4-year time span, except for the absence of other official stroke awareness education, which could potentially influence the participants’ responses and overall performance. Finally, it should be noted that during the third phase of the study, 6 months after the initial program implementation, many participants abstained due to the acute phase of the COVID-19 pandemic at that time.

To enhance the generalizability of our findings, future research should include larger and more diverse participant groups, encompassing various socioeconomic backgrounds and geographical regions. This broader sampling would provide a more comprehensive understanding of the program’s effectiveness across different populations.

Additionally, while our study provides a 4-year follow-up, it is essential to consider the impact of external factors such as media exposure and individual experiences on participants’ knowledge retention. Future studies should incorporate more detailed assessments of these external influences, examining how they affect the long-term sustainability of knowledge gained through the program.

Moreover, although our research highlights the positive outcomes of the FAST Heroes campaign, a more robust evaluation of its impact on actual emergency response behaviors is needed. Future studies should investigate whether the program leads to increased emergency calls made to services during stroke incidents. This could be achieved through collaboration with emergency service providers to track and analyze call data, providing a clearer picture of the program’s practical implications and its effectiveness in real-world scenarios.

By addressing these areas, future research can significantly contribute to the ongoing development and refinement of the FAST Heroes program.

## 6 Conclusion

In summary, FAST Heroes seems to have resulted in a sustained enhancement of participants’ awareness regarding the common signs of a stroke and the importance of promptly contacting emergency services following a stroke. However, this effect was somewhat diminished over the long term, which is rather justifiable since no other education has intervened nearly 4 years after the initial implementation of this program. Thus, it is reasonable to consider that sustaining the achieved progress may require some brief follow-up educational sessions.

## Data Availability

The raw data supporting the conclusions of this article will be made available by the authors, without undue reservation.
