# Supplementary material for: Post-implementation knowledge retention of stroke: the lasting influence of FAST Heroes
Source: Front Public Health. 2024 Oct 1;12:1400409. doi: 10.3389/fpubh.2024.1400409 (PMC11473299; doi:10.3389/fpubh.2024.1400409)
Supplement: Supplementary file 1 [file Data_Sheet_1.docx]

*Questionnaire utilized in Phase 4*

(1) ‘Are you familiar with the “FAST Heroes” program?’

(2) ‘What are the main symptoms of a stroke?’

(3) ‘What is the most appropriate number used to call an ambulance in case of a stroke?’

(4) ‘What should I do if someone suddenly has a severe headache?’

(5) ‘What should I do if someone suddenly isn't speaking clearly or is slurring their words?’

(6) ‘What should I do if someone suddenly feels weak and dizzy?’

(7) ‘What should I do if someone suddenly can't move one of their arms?’

(8) ‘What should I do if I see someone's face suddenly drooping on one side?’

(9) What should I do if someone suddenly feels chest pain?

Questions 4 to 9 aim to assess participants' understanding of stroke symptoms by evaluating their ability to differentiate between symptoms related to strokes, which had been taught through the FAST Heroes program, and those unrelated to strokes and/or not covered during the program. Thus, Questions (4), (6) and (9) were used as a distractor.
